# Low anterior resection syndrome: Incidence and association with quality of life

**DOI:** 10.1002/ags3.12724

**Published:** 2023-08-02

**Authors:** Yuko Homma, Toshiki Mimura, Koji Koinuma, Hisanaga Horie, Alan Kawarai Lefor, Naohiro Sata

**Affiliations:** ^1^ Department of Surgery, Division of Gastroenterological, General and Transplant Surgery Jichi Medical University Tochigi Japan

**Keywords:** incidence, low anterior resection syndrome, quality of life, rectal tumor, risk factor

## Abstract

**Aim:**

Low anterior resection syndrome (LARS) causes devastating symptoms and impairs quality of life (QOL). Although its incidence and risk factors have been reported, these data are scarce in Japan. This study aimed to elucidate the incidence and risk factors of LARS as well as to evaluate its association with QOL in Japanese patients.

**Method:**

Patients with anal defecation at the time of the survey between November 2020 and April 2021 were included, among those who underwent anus‐preserving surgery for rectal tumors between 2014 and 2019 in tertiary referral university hospital. The severity of LARS and QOL were evaluated with the LARS score and the Japanese version of the fecal incontinence quality of life scale (JFIQL), respectively. Primary endpoint was the incidence of major LARS. Secondary endpoints were risk factors and association with JFIQL.

**Results:**

Of 332 eligible patients, 238 (71.7%) answered the LARS survey completely. The incidence of major LARS was 22% overall, and 48% when limited to lower tumors. Independent risk factors included lower tumors (OR: 7.0, 95% CI: 2.1–23.1, *p* = 0.001) and surgical procedures with lower anastomoses (OR: 4.6, 95% CI: 1.2–18.5, *p* = 0.03). The JFIQL generic score correlated moderately with the LARS score (correlation coefficient of −0.65). The JFIQL generic score was also significantly lower in lower tumors.

**Conclusions:**

The incidence of major LARS is 22% in Japanese patients, and independent risk factors include lower tumors and surgical procedures with lower anastomoses. More severe LARS is associated with worse QOL which is significantly more impaired in patients with lower tumors.

## Graphical abstract

This cross‐sectional survey study has investigated the incidence and risk factors of low anterior resection syndrome (LARS) as well as evaluated its association with QOL in Japanese patients. The incidence of major LARS is 22%, and independent risk factors include lower tumors and surgical procedures with lower anastomoses. More severe LARS is associated with worse QOL which is significantly more impaired in patients with lower tumors.

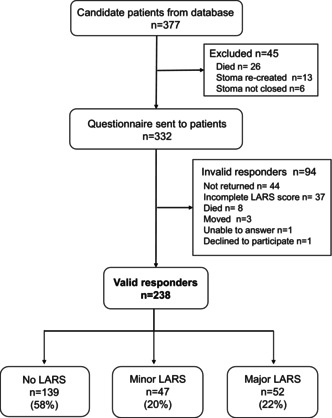

## 1 INTRODUCTION

Patients after rectal resection have various symptoms, collectively referred to as low anterior resection syndrome (LARS). The severity is usually evaluated with the LARS score, developed by Emmertsen et al.^1^ and consisting of five questions including incontinence to flatus, incontinence to liquid stool, frequency of bowel movements, clustering of stools and urgency. The severity is categorized into no, minor, and major LARS depending on the score. According to a meta‐analysis of 11 studies by Croese et al. in 2018,^2^ the incidence of major LARS was 41%. A low tumor height and radiotherapy were reported as risk factors for LARS. More studies have subsequently reported the incidence and risk factors for LARS from several countries,^3^, ^4^ but are scarcely reported from Japan.^5^

Various LARS symptoms severely affect quality of life (QOL) in patients with LARS.^6^ Patient QOL has previously been evaluated with questionnaires including the European Organization for Research and Treatment of Cancer Quality of Life Questionnaire Core Module (EORTC QLQ‐C30),^7^ Fecal Incontinence Quality of Life scale (FIQL),^8^ EuroQol five‐dimension (EQ‐5D),^9^ and others. However, few studies have evaluated the association of LARS symptoms with QOL in Japanese patients after anus‐preserving surgery (APS).^10^

This study aimed to elucidate the incidence and risk factors for LARS as well as to evaluate its association with QOL in Japanese patients after APS for rectal tumors.

## 2 METHODS

### 2.1 Patients

Candidates included patients who underwent APS for rectal tumors between January 1, 2014, and December 31, 2019. Patients were identified from a prospectively maintained database. Questionnaires were sent by postal mail, excluding patients who had stomas or had died according to the medical record. Patients included for analysis were valid responders who answered the LARS score survey completely. Patients who were invalid responders, had died, moved, did not return the questionnaire, or returned an incomplete LARS score were excluded from analysis.

### 2.2 Parameters to evaluate LARS and QOL

Incidence and severity of LARS symptoms were assessed using the LARS score ranging from 0 to 42, categorized into “no LARS (0‐20),” “minor LARS (21‐29),” and “major LARS (30‐42).”^1^ To evaluate QOL, the Japanese version of the fecal incontinence quality of life scale (JFIQL) was used.^11^ The JFIQL is a validated Japanese version of the original FIQL (in English),^12^ which is a fecal incontinence‐specific QOL questionnaire. The FIQL includes 29 questions in four domains, including Lifestyle, Coping/Behavior, Depression/Self‐perception, and Embarrassment. Each item is rated from 1 to 6 points depending on the question and includes a “not applicable (N/A) option” if the question is not applicable to the patient. The generic score is the average response to answered items among 29 questions and ranges from 1 to 4.1 with higher score meaning better QOL. If more than half of the items are unanswered, the patient is excluded from analysis due to missing values. “N/A” is also regarded as a missing value. The rules of the generic score are also applied to the four domains.

### 2.3 Study design and endpoints

Questionnaires were distributed and collected from November 2020 to April 2021. Clinical and surgical data were compared between valid and invalid responders, including age, gender, comorbidities, type of rectal tumor, tumor location and neoadjuvant treatment. Tumor location was classified as the upper, middle, or lower rectum, according to the Japanese classification of colorectal carcinoma. The landmarks for the classification are sacral promontory, inferior border of the second sacral vertebra, peritoneal reflection, and the upper border of anal canal, which divide the rectum into the three parts. Surgical data included type of surgical procedure, type of mesorectal excision (ME), type of anastomosis, presence of diverting stoma, and other data. Type of surgical procedure included high anterior resection (HAR), low anterior resection (LAR), ultra‐low anterior resection (uLAR), and intersphincteric resection (ISR). For HAR, the anastomosis is above the peritoneal reflection. For LAR, the anastomosis is between the peritoneal reflection and 1 cm above the anorectal junction. For uLAR, the anastomosis is within 1 cm above anorectal junction. For ISR, the internal anal sphincter is partially or totally resected.

The primary endpoint was the incidence of LARS, defined as the proportion of major LARS among all valid responders, because Emmertsen et al.^6^ reported that major LARS had a significant impact on QOL while minor and no LARS had minimal impact on QOL. Secondary endpoints included risk factors for major LARS as well as the association between LARS and QOL. To identify risk factors for major LARS, the clinical and surgical data were compared between patients with no LARS and those with major LARS using multivariate analysis.

To evaluate the association between LARS and QOL, the JFIQL result was compared among the three groups including “no LARS,” “minor LARS,” and “major LARS.” The association between LARS scores and JFIQL was also evaluated with correlation analysis.

### 2.4 Statistical analysis

Data were regarded as non‐parametric, and values expressed as median and range. For comparison of the two groups, the Mann–Whitney U test was used for continuous data, while Fisher's exact probability test or chi‐square test was used for categorical data accordingly. For multivariate analysis, only factors with *p* values < 0.05 on univariate analysis were included to calculate odds ratios (OR), 95% confidence intervals (CI), and *p* values. Correlation coefficients were calculated using Spearman correlation analysis to evaluate the association between LARS scores and JFIQL.

GraphPad Prism 8.0 (GraphPad Software Inc.) software was used for all statistical analyses except multivariate analyses, for which SPSS version 23 (IBM Inc.) was used. *p* values less than 0.05 were regarded as statistically significant.

## 3 RESULTS

### 3.1 Process of patient selection

**FIGURE 1:**
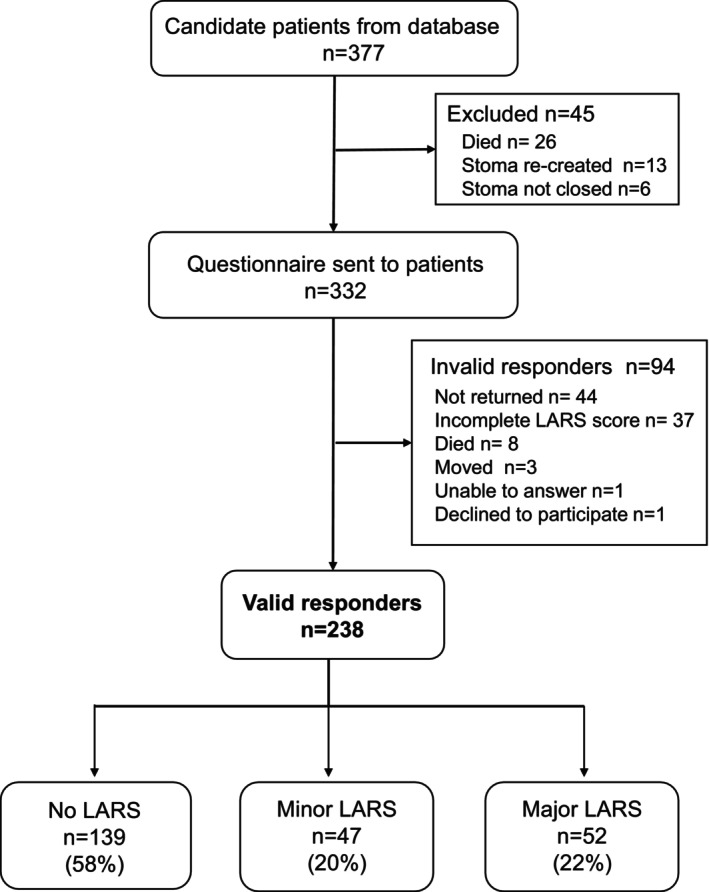
Flowchart of patient selection. LARS, low anterior resection syndrome.

Questionnaires were sent to 332 eligible patients out of 377 candidates identified in the database, excluding 45 who died, had a stoma re‐created or stoma not closed (Figure 1). Of these 332 patients, 238 (71.7%) were valid responders who answered the LARS questionnaire completely, while 94 were invalid responders for reasons shown in Figure 1.

Demographic data were compared between the 238 valid responders and 93 invalid responders. One invalid responder was excluded because of unwillingness to participate. Demographic data were comparable between the two groups except for age, central nervous system comorbidities, and the presence of psychiatric disorders (Table 1). Invalid responders were significantly older than valid responders, while the proportion with central nervous system and psychiatric disorders was significantly higher in invalid responders than in valid responders.

**TABLE 1:** Demographic data: Valid versus invalid responders.

Baseline variables	Valid responders (*n* = 238)		Invalid responders (*n* = 93)		*p* Value
Age					
Median (range)	67	(29–91)	70	(31–89)	0.01*
Gender					
Male	149	(63%)	54	(58%)	0.45**
Female	89	(37%)	39	(42%)	
Comorbidities					
Diabetes mellitus	43	(18%)	20	(22%)	0.53**
Hypertension	96	(40%)	23	(25%)	0.22**
Coronary disease	10	(4%)	5	(5%)	0.77**
Spinal cord impairment	11	(5%)	4	(4%)	>0.99**
Central nervous system disorder	7	(3%)	12	(12%)	0.001**
Psychiatric disorder	2	(1%)	7	(8%)	0.003**
Type of rectal tumor					
Adenocarcinoma	230	(97%)	92	(99%)	0.50***
Neuroendocrine tumor	7	(2.6%)	1	(1%)	
Benign tumor	1	(0.4%)	–	–	
Tumor location					
Upper	82	(34.5%)	32	(34%)	0.31***
Middle	74	(31%)	36	(39%)	
Lower	82	(34.5%)	25	(27%)	
Neoadjuvant CRT	24	(10%)	5	(5%)	0.20**
Surgical procedures for rectal resection					
HAR	84	(35%)	37	(40%)	0.58***
LAR	97	(41%)	38	(41%)	
uLAR	50	(21%)	14	(15%)	
ISR	7	(3%)	4	(4%)	
Type of ME					
PME	181	(76%)	75	(81%)	0.47**
TME	57	(24%)	18	(19%)	
Type of anastomosis					
DST	221	(93%)	89	(96%)	0.45**
Hand sewn	17	(7%)	4	(4%)	
Diverting stoma	78	(33%)	25	(27%)	0.69**
Ileostomy	72		22		
Colostomy	6		3		
Anastomotic leak	5	(2%)	3	(3%)	0.69**
Stage (according to UICC 8th edition)					
0	2	(1%)	–	–	0.23***
I	100	(42%)	35	(38%)	
II	51	(21%)	29	(31%)	
III	78	(33%)	24	(26%)	
IV	6	(3%)	5	(5%)	
Adjuvant chemotherapy	71	(30%)	21	(23%)	0.22**
Interval since rectal resection or diverting stoma closure					
Months, median (range)	44.7	(3.0–81.5)	46.4	(10.7–82.0)	0.41*

*Note*: *Mann–Whitney U test; **Fisher's exact test; ***Chi‐square test.

Bold *p* values mean statistical significance.

Abbreviations: CRT, chemoradiotherapy; DST, double stapling technique; HAR, high anterior resection; ISR, intersphincteric resection; LAR, low anterior resection; ME, mesorectal excision; NA, not available; PME, partial ME; TME, total ME; uLAR, ultra‐low anterior resection.

### 3.2 Demographic data of valid responders

The 238 valid responders were the subjects for analysis in this study. Their median age was 67 years old, and 149 (63%) were male (Table 1). Tumor location was evenly distributed among the upper, middle, and lower rectum. Neoadjuvant chemoradiotherapy (CRT) was administered to only 10%. LAR was most commonly performed procedure for rectal resection at 41% and ISR was least at 3%. All patients underwent straight anastomosis. A diverting stoma was created in 33%. Anastomotic leakage occurred in only five patients (2%). The interval since rectal resection or diverting stoma closure was a median of 44.7 months.

### 3.3 Incidence of LARS

The median LARS score was 16 (range:0–41) among the 238 valid responders. As shown in Figure 1, the incidence of no, minor, and major LARS was 58%, 20%, and 22%, respectively. According to the definition for this study, the incidence of LARS was 22%, being major LARS.

### 3.4 Risk factors for developing major LARS

A univariate analysis was conducted to identify risk factors for major LARS. As shown in Table 2, clinical and surgical parameters were compared between the no LARS and major LARS groups. A significant difference was found for eight factors including age, tumor location, neoadjuvant CRT, surgical procedure, type of ME, type of anastomosis, presence of diverting stoma, and interval since rectal resection or diverting stoma closure. Patients with major LARS were significantly younger than those with no LARS. Patients with major LARS had significantly lower tumors than those with no LARS. The major LARS rate was highest with lower rectal lesions (48%), followed by middle (12%) and upper rectal tumors (5%). A significantly higher proportion of those with major LARS (33%) received neoadjuvant CRT compared to those with no LARS (5%). Surgical procedures with lower anastomoses were significantly more common among those with major LARS compared to no LARS, reflecting tumor location, except ISR, which was probably due to the small number of patients. The proportion undergoing TME was significantly higher in those with major LARS (52%) compared to those with no LARS (13%). The proportion of hand sewn anastomoses was significantly higher among those with major LARS (21%) compared to no LARS (3%). A significantly higher proportion of patients with major LARS (63%) underwent diverting stoma creation than those with no LARS (22%). The interval since rectal resection or diverting stoma closure was significantly shorter in those with major LARS than those with no LARS.

**TABLE 2:** Univariate analysis: comparison of no LARS and major LARS groups.

Compared factors	No LARS (*n* = 139)		Major LARS (*n* = 52)		Major LARS rate	*p* Value
Age						
Median (range)	69	(44–91)	63	(29–91)	–	0.008*
Gender						
M	82	(59%)	35	(67%)	23%	0.32**
F	57	(41%)	17	(33%)	19%	
Comorbidities						
Diabetes mellitus	26	(19%)	9	(17%)	21%	>0.99**
Hypertension	60	(43%)	19	(37%)	20%	0.51**
Coronary disease	8	(6%)	1	(2%)	10%	0.45**
Spinal cord impairment	6	(4%)	2	(4%)	18%	>0.99**
Central nervous system disorder	5	(4%)	1	(2%)	14%	>0.99**
Psychiatric disorder	1	(1%)	1	(1%)	50%	0.47**
Type of rectal tumor						
Adenocarcinoma	136	(98%)	49	(94%)	21%	0.21***
Neuroendocrine tumor	2	(1%)	3	(6%)	43%	
Benign tumor	1	(1%)	–	–	–	
Tumor location						
Upper	69	(50%)	4	(8%)	5%	<0.0001***
Middle	47	(34%)	9	(17%)	12%	
Lower	23	(17%)	39	(75%)	48%	
Neoadjuvant CRT						
Yes	7	(5%)	17	(33%)	71%	0.004**
No	132	(96%)	35	(67%)	16%	
Surgical procedures for rectal resection						
HAR	70	(50%)	3	(6%)	4%	<0.0001***
LAR	53	(38%)	22	(42%)	23%	
uLAR	13	(9%)	25	(48%)	50%	
ISR	3	(2%)	2	(4%)	29%	
Type of ME						
PME	123	(88%)	25	(48%)	14%	<0.0001**
TME	16	(13%)	27	(52%)	47%	
Type of anastomosis						
DST	135	(97%)	41	(79%)	19%	0.0001**
Hand sewn	4	(3%)	11	(21%)	65%	
Diverting stoma						
Yes	31	(22%)	33	(63%)	42%	<0.0001**
No	108	(78%)	19	(37%)	12%	
Anastomotic leak	2	(1%)	1	(3%)	20%	>0.99**
Stage: (according to UICC 8th edition)						
0	1	(1%)	1	(2%)	50%	0.68***
I	58	(42%)	19	(37%)	19%	
II	27	(19%)	15	(29%)	29%	
III	47	(34%)	16	(31%)	21%	
IV	5	(4%)	1	(2%)	17%	
No malignant	1	(1%)	–	–	–	
Adjuvant chemotherapy	43	(31%)	15	(29%)	21%	0.86**
Interval since rectal resection or diverting stoma closure						
Months, median (range)	46.3	(10.7–81.5)	35.3	(3.0–80.8)	–	0.013*

*Note*: *Mann–Whitney U test; **Fisher's exact test; ***Chi‐square test.

Bold *p* values mean statistical significance.

Abbreviations: CRT, chemoradiotherapy; DST, double stapling technique; HAR, high anterior resection; ISR, intersphincteric resection; LAR, low anterior resection; ME, mesorectal excision; NA, not available; PME, partial ME; TME, total ME; uLAR, ultra‐low anterior resection.

Multivariate analysis revealed that lower tumors (OR: 7.0, 95% CI: 2.1–23.1, *p* = 0.001) and surgical procedures other than HAR (i.e. LAR + uLAR + ISR) (OR: 4.6, 95% CI: 1.2–18.5, *p* = 0.03) were independent risk factors for major LARS among the eight factors identified by univariate analysis (Table 3).

**TABLE 3:** Multivariate analysis: comparison of no LARS and major LARS groups.

		Odds ratio	95% confidence interval	*p* Value
Age	<65yo vs. 65≤	0.5	0.2–1.1	0.10
Tumor location	Upper + Middle vs. Lower	**7.0**	**2.1–23.1**	**0.001**
Neoadjuvant CRT	Yes vs. No	3.0	0.8–10.6	0.10
Surgical procedures for rectal resection	HAR vs. LAR + uLAR + ISR	**4.6**	**1.2–18.5**	**0.03**
Mesorectal excision	PME vs. TME	1.2	0.3–4.2	0.82
Type of Anastomosis	DST vs. Hand sewn	2.1	0.5–9.5	0.33
Diverting stoma	Yes vs. No	0.5	0.1–1.9	0.33
Interval since rectal resection or diverting stoma closure	<12 months vs. 12≤	4.2	0.7–23.1	0.10

*Note*: Bold *p* values mean statistical significance, indicating the items are independent risk factors for Major LARS.

Abbreviations: DST, double stapling technique; HAR, high anterior resection; ISR, intersphincteric resection; LAR, low anterior resection; PME, partial mesorectal excision; TME, total mesorectal excision; uLAR, ultra‐low anterior resection.

### 3.5 The association between LARS and QOL

Of 238 valid responders, seven were excluded from further analysis based on the generic score of the JFIQL due to missing values because they did not answer more than half of the items. Further, another 70 patients were excluded from analysis due to their answer of “not apply,” regarded as a missing value. Therefore, the remaining 161 patients (67.6%) were included in the generic score analysis. The median generic score of the JFIQL (*n* = 161) was 3.4 (range:1.2–4.1). Similarly, for the four domains, the median score was 3.6 (range:1–4) in Lifestyle (*n* = 152), 3.1 (range:1–4) in Coping/Behavior (*n* = 169), 3.8 (range:1.1–4.5) in Depression/Self‐perception (*n* = 154), and 3.7 (range:1–4) in Embarrassment (*n* = 159).

**FIGURE 2:**
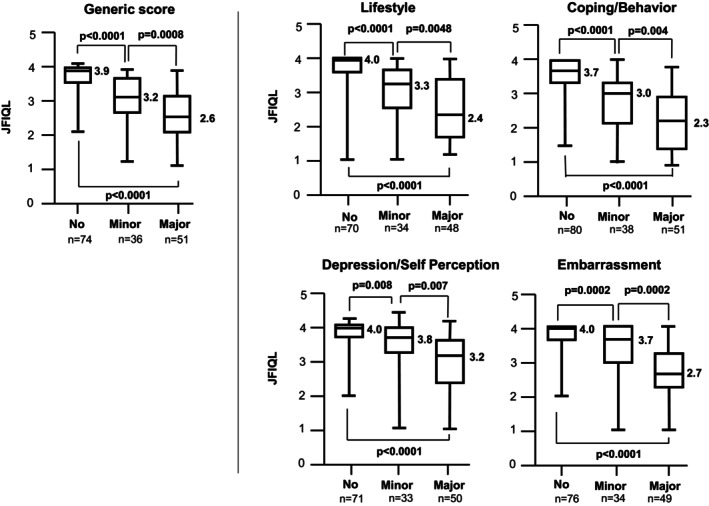
Comparison of JFIQL scores among “no LARS,” “minor LARS,” and “major LARS” groups. LARS: low anterior resection syndrome; JFIQL: Japanese version of the fecal incontinence quality of life scale.

As shown in Figure 2, the association between QOL and LARS was evaluated by comparing the JFIQL scores among the “no LARS,” “minor LARS,” and “major LARS” groups. In the generic score as well as all four domains, scores were significantly lower in the order of “major,” “minor,” and “no” LARS groups, demonstrating that more severe LARS was associated with worse QOL.

**FIGURE 3:**
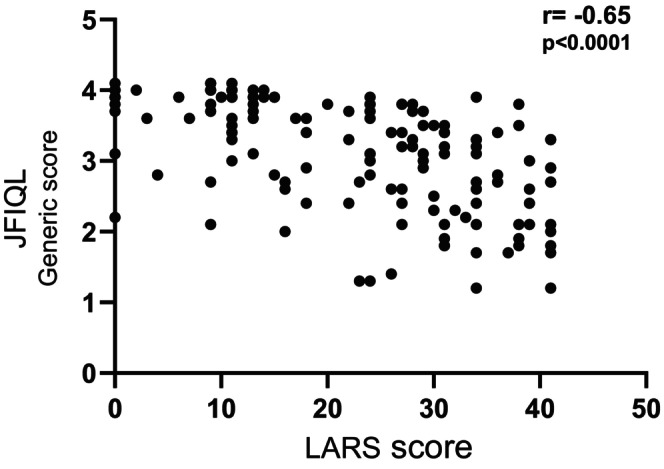
Correlation between LARS score and JFIQL generic score. LARS: low anterior resection syndrome, JFIQL: Japanese version of the fecal incontinence quality of life scale.

In correlation analysis, the LARS score moderately correlated with the generic score of the JFIQL with a correlation coefficient of −0.65 (Figure 3). Therefore, this also shows that more severe LARS was associated with worse QOL.

### 3.6 LARS severity and QOL depending on tumor location

**FIGURE 4:**
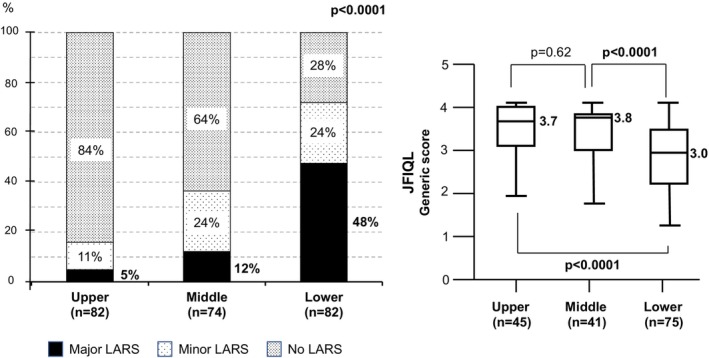
LARS severity (left panel) and QOL (right panel) according to tumor location. JFIQL: Japanese version of the fecal incontinence quality of life scale; LARS, low anterior resection syndrome; QOL, quality of life.

Since tumor location was identified as an independent risk factor for LARS, its association with LARS severity and QOL was further analyzed. As shown in Figure 4, the proportion of major LARS was significantly higher for patients with lower rectal lesions, followed by middle and upper tumors (48% vs. 12% vs. 5%, *p* < 0.0001). The JFIQL generic score was significantly lower for lower tumors (median 3.0, range: 1.2–4.1, *p* < 0.0001), compared with middle (3.8: 1.8–4.1) and upper tumors (3.7: 1.9–4.1), indicating that QOL was significantly more impaired in patients with lower tumors.

## 4 DISCUSSION

This study shows that the incidence of major LARS is 22% in Japanese patients after APS for rectal tumors. Among patients with lower tumors, it is much higher at 48%. The independent risk factors for major LARS include lower rectal tumors and undergoing a surgical procedure (LAR, uLAR, and ISR) other than HAR. This study also demonstrates that QOL is more significantly impaired in patients with more severe LARS and lower tumors. This is the first study from Japan that has demonstrated the significant association between LARS score and QOL.

These findings are useful when discussing planned surgical procedures with patients who will undergo resection of rectal tumors. In particular, patients with lower tumors face difficult decisions—whether to undergo APS or an abdominoperineal resection (APR) with permanent colostomy. Many patients find the idea of a permanent colostomy unacceptable, whilst other patients find that APS is associated with remarkably impaired bowel function and worse QOL, as shown in the present study. These decisions should be made and shared by thorough discussion between patients and their attending surgeons, a process called shared decision‐making.^13^ The results of this study will serve as useful decision aids in this shared decision‐making.

In patients who choose APS, the results of the present study allow them to anticipate the probability and severity of LARS preoperatively. According to the present study, 48% of patients with lower tumors will suffer from major LARS, and their QOL will be significantly impaired compared to patients with middle and higher tumors. However, it should be explained preoperatively that LARS is treatable using various modalities including medications, biofeedback, transanal irrigation, sacral neuromodulation, and others.^14^ These therapies could improve the QOL of patients with LARS. If all treatments fail or are not indicated, even a permanent stoma is an option after trying APS.

Three meta‐analyses have reported the incidence of LARS and its risk factors as shown in Table 4. Croese et al.^2^ reported that the incidence of major LARS was 41% in their meta‐analysis of 11 studies. In that report, low tumor height and radiotherapy were suggested as consistent risk factors for developing major LARS. Sun et al.^15^ also reported in their meta‐analysis of 36 studies that the pooled incidence of major LARS was 44%. Independent risk factors for major LARS included TME (reflecting tumor location), long‐course neoadjuvant radiotherapy, anastomotic leakage, and a diverting stoma. Ye et al.^16^ reported in a meta‐analysis of 21 studies that the incidence of major LARS was 49.7% and risk factors for major LARS included low tumor height, low anastomotic height, radiotherapy and chemotherapy, anastomotic leakage, and diverting stoma.

**TABLE 4:** Comparison of the present study with three previous meta‐analyses.

Authors (reported year)	Major LARS rate	Risk factors	Rate of lower tumor	Rate of TME	Rate of radiotherapy	Rate of diverting stoma	Rate of anastomotic leakage
Croese et al.^2^ (2018)	41% (95% CI, 34%–48%)	Low tumor height Radiotherapy	–	59%–72%	20%–78%	32%–55%	–
Sun et al.^15^ (2021)	44% (95% CI:40%–48%)	TME Long‐course neoadjuvant radiotherapy Anastomotic leakage Diverting stoma	–	74% (2270/3078)	46% (2938/6452)	–	–
Ye et al.^16^ (2022)	49.7%	Low tumor height Low anastomotic height Radiotherapy and chemotherapy Anastomotic leakage Diverting stoma.	54% (307/686)	–	15% (176/1196)	42% (393/948)	7% (133/1896)
Present study	22%	Lower tumor Surgical procedures with lower anastomosis	34.5%	24%	10%	33%	2%

Abbreviations: CI, confidence interval; LARS, low anterior resection syndrome; TME, total mesorectal excision.

In the present study, the incidence of major LARS is 22%, which is remarkably lower than over 40% as reported in the three previous meta‐analyses. This difference can be attributed to differences in the rate of lower tumors between the present study and the meta‐analyses. The lower tumor rate in the present study is 34.5%, which is considerably lower than 54% reported by Ye et al.^16^ It is known that lower tumors require higher rates of TME, radiotherapy, and diverting stomas, with tumor location being related to the rates of these therapeutic modalities. As is shown in Table 4, the rates of TME and radiotherapy are remarkably lower, and the rate of diverting stoma is slightly lower in the present study than in the meta‐analyses. This may indirectly prove that the rate of lower tumors is remarkably lower in the present study than in the three meta‐analyses. However, if the tumor location is confined to lower tumors in the present study, the rate of major LARS is 48%, which is similar to the over 40% shown in the three meta‐analyses. Additionally, the difference in rate of developing major LARS may be influenced by differences in the rate of anastomotic leaks, which is slightly lower in the present study at 2% compared to 7% reported by Ye et al.^16^

In the present study, the independent risk factors for developing major LARS include lower tumors and a surgical procedure other than HAR. However, TME, radiotherapy, creating a diverting stoma and anastomotic leakage are not identified as independent risk factors in the present study, although they were reported to be risk factors in the meta‐analyses as shown in Table 4. This difference might be also attributed to differences in the rate of lower tumors in the present study. Since the proportion of patients who have these risk factors is relatively lower in the present study than in the meta‐analyses, they are more unlikely to be identified as independent risk factors. If only lower tumors were analyzed in the present study, they might be identified as independent risk factors for developing major LARS. This hypothesis will be investigated in a future study.

It is well‐known that LARS negatively impacts QOL after APS for rectal tumors.^6^ Al Rashid et al.^17^ reported a systematic review of 18 studies evaluating the correlation between symptomatic and QOL scores in patients with LARS. In their review, only two studies used FIQL as the QOL score, while other studies used EORTC QLQ‐C30, EQ‐5D, SF‐36, or Psychological General Well‐Being Index. The two studies that used FIQL correlated it with Fecal Incontinence Severity Index or “Self‐reported fecal incontinence within the last week,” while in this study, the Japanese FIQL (JFIQL) is correlated with LARS score. In the present study, the generic score as well as the four domain scores of the JFIQL are significantly lower in the order of “major,” “minor,” and “no” LARS groups, demonstrating that more severe LARS is associated with worse QOL. The present study also shows that the LARS score correlates moderately with the generic score of JFIQL. A significant correlation between LARS score and FIQL has not been reported according to the systematic review by Al Rashid et al. This is the first study from Japan that has demonstrated the significant association between LARS score and JFIQL. Recently, however, Kim et al.^18^ from Korea also reported similar results, regarding the association between LARS score and FIQL.

There are two limitations in this study. First, the results of the present study might not represent all patients who underwent APS, because significant differences are found between valid and invalid responders, regarding age and comorbidities including central nervous system and psychiatric disorders. This limitation might be inherent to the study design of a self‐administered questionnaire, because older patients or those with these comorbidities may have difficulty responding to the questionnaires. Second, the QOL results in the present study might not represent all patients with LARS, because JFIQL scores are evaluated in about 70% of 238 valid responders. Many patients are excluded from this analysis due to their answer of “not apply,” regarded as missing values.

In conclusion, this study has elucidated that the incidence of major LARS is 22% in Japanese patients after anus‐preserving surgery for rectal tumors. Especially in those with lower tumors, it is much higher at 48%. The independent risk factors for major LARS include lower rectal tumors and surgical procedures with lower anastomoses. This study also demonstrates that QOL is more significantly impaired in patients with more severe LARS and lower tumors. These findings can be used as decision‐making aids for shared decision‐making when discussing surgical procedures with patients who will undergo rectal resection.

## AUTHOR CONTRIBUTIONS

YH contributed to study design, data collection and analysis as well as drafting and editing the article. TM contributed to data analysis and revising the article. AL contributed to revising the article, particularly as a native English‐speaking medical editor. All authors read and approved the final article.

## FUNDING INFORMATION

There is no funding.

## CONFLICT OF INTEREST STATEMENT

The authors declare no conflicts of interest for this article.

## ETHICS STATEMENT

Approval of the research protocol: This study protocol was approved by the Jichi Medical University Central Clinical Research Ethics Committee (approval number 20–039).

Informed Consent: Written informed consent was obtained from all participants.

Registry and the registration No. of the study/trial: N/A.

Animal Studies: N/A.
